# Lead Contamination in Cocoa and Cocoa Products: Isotopic Evidence of Global Contamination

**DOI:** 10.1289/ehp.8009

**Published:** 2005-05-26

**Authors:** Charley W. Rankin, Jerome O. Nriagu, Jugdeep K. Aggarwal, Toyin A. Arowolo, Kola Adebayo, A. Russell Flegal

**Affiliations:** 1 Environmental Toxicology, WIGS, University of California, Santa Cruz, California, USA; 2 Department of Environmental Health Sciences, School of Public Health, University of Michigan, Ann Arbor, Michigan, USA; 3 Earth Sciences, University of California, Santa Cruz, California; 4 Department of Environmental Management and Toxicology, and; 5 Department of Agricultural Extension and Rural Development, University of Agriculture, Abeokuta, Nigeria

**Keywords:** chocolate, cocoa, contamination, isotopes, lead, natural foods

## Abstract

In this article we present lead concentrations and isotopic compositions from analyses of cocoa beans, their shells, and soils from six Nigerian cocoa farms, and analyses of manufactured cocoa and chocolate products. The average lead concentration of cocoa beans was ≤ 0.5 ng/g, which is one of the lowest reported values for a natural food. In contrast, lead concentrations of manufactured cocoa and chocolate products were as high as 230 and 70 ng/g, respectively, which are consistent with market-basket surveys that have repeatedly listed lead concentrations in chocolate products among the highest reported for all foods. One source of contamination of the finished products is tentatively attributed to atmospheric emissions of leaded gasoline, which is still being used in Nigeria. Because of the high capacity of cocoa bean shells to adsorb lead, contamination from leaded gasoline emissions may occur during the fermentation and sun-drying of unshelled beans at cocoa farms. This mechanism is supported by similarities in lead isotopic compositions of cocoa bean shells from the different farms (^206^Pb/^207^Pb = 1.1548–1.1581; ^208^Pb/^207^Pb = 2.4344–2.4394) with those of finished cocoa products (^206^Pb/^207^Pb = 1.1475–1.1977; ^208^Pb/^207^Pb = 2.4234–2.4673). However, the much higher lead concentrations and larger variability in lead isotopic composition of finished cocoa products, which falls within the global range of industrial lead aerosols, indicate that most contamination occurs during shipping and/or processing of the cocoa beans and the manufacture of cocoa and chocolate products.

Lead contamination in candies is a longstanding problem that has evolved with time. Fred Accum (1820) was the first person to systematically investigate the widespread contamination of confectionaries with metallic poisons. His study of 100 sweets sold in Britain during the early part of the 19th century found that 59 contained lead chromate, 12 contained red lead, and 10 contained Brunswick green (a mixture of Prussian blue and lead chromate). Most of the lead observed at that time was attributed to intentional adulteration or wraps that were glazed, colored, or printed with lead compounds. Since the middle of the 19th century, various measures including regulations and public education were implemented to minimize the contamination of candies from such sources (Nriagu 1985). Today, industrial activities dominate the global flux of lead in the environment (Flegal and Smith 1995; Nriagu and Pacyna 1988) and have become the predominant sources of contaminant lead in many food items, including candies. This remains true despite recent measures taken to reduce environmental lead contamination and to minimize human exposure to lead that have lowered the concentrations of this metal in foods and human populations (Egan 2002; Pirkle et al. 1998; Thomas et al. 1999; von Storch et al. 2003).

Specific focus on the source of lead in cocoa, the principal material used to make chocolate, began during the late 1970s. Despite subsequent marked reduction in the release of lead into the environment, due primarily to removal of lead from gasoline (Nriagu 1990), recent market-basket surveys still indicate continued lead contamination in some foods, notably manufactured cocoa and chocolate products. For example, in the 2000 U.S. Food and Drug Administration (FDA) Total Diet Survey (TDS), the average lead content for milk chocolate candy bars (27 ng/g) was the fourth highest reported for all food items (FDA 2000). This observation was corroborated by both the 20th Australian TDS, where milk chocolate had the second highest value of 65 foods, with a mean value of 21 ng/g and a maximum value of 40 ng/g (Food Standards Australia New Zealand 2003), and the 1997/1998 New Zealand TDS report, where the lead concentration in chocolate biscuits (15 ng/g) was 3-fold greater than those of cracker (5.2 ng/g) and plain sweet (5.2 ng/g) biscuits (Vannoort et al. 2000). In a recent study of cocoa-based chocolates sold in India, Dahiya et al. (2005) found the average lead concentrations to be 1.92 μg/g (range, 0.05–8.3 μg/g), and Onianwa et al. (1999) found the average lead content of cocoa powders sold in Nigeria to be 310 ng/g with a range of 80–880 ng/g.

The latter measurements are consistent with reports of elevated levels of lead in cocoa by the Cocoa Producer’s Alliance (COPAL), which is based in Nigeria. COPAL is the supplier of 75% of all cocoa beans to the world market (COPAL 2004a). The sources of lead in Nigerian cocoa products, which have become a concern, may conceivably include lead from local soils and rocks where the cocoa plant is grown; farming practices (e.g., the application of fertilizers, lead-containing pesticides, composts and other soil additives); atmospherically deposited lead; handling and processing of cocoa beans after harvesting (including drying in open air, transportation, and storage); grinding and manufacturing processes (wear and tear of lead-soldered machine parts); mixtures and additives to final products; and packaging and wrapping material.

The presence of relatively high concentrations in a consumer product that is heavily marketed to children is a special concern, because children are particularly susceptible to lead poisoning (Silbergeld 1997). The maximum permissible level (MPL) of lead recently proposed by Codex Alimentarius Commission was 0.1 μg/g for cocoa butter (a key ingredient in chocolate) and 1.0 μg/g for cocoa mass and cocoa powder (COPAL 2004b). In India, the lead content of chocolates (1.92 μg/g) exceeds the MPL for cocoa powder and cocoa butter. The provisional tolerable weekly intake (PTWI) of lead has been set at 25 μg/kg body weight for children [World Health Organization (WHO) 1993], equivalent to 3.6 μg/kg body weight/day. Examination of labels on various chocolate powders sold in the United States show that the serving sizes are typically in the range of 15–25 g. Assuming an absorption of 40% [Agency for Toxic Substances and Disease Registry (ATSDR) 1999], by consuming cocoa powder of average serving size (20 g) with an average lead content of 0.2 μg/g, a child weighing 15 kg would be acquiring approximately 3% of his or her PTWI from this source. By consuming cocoa powder with the maximum lead content (0.79 μg/g) reported by Mounicou et al. (2003), a child would receive about 12% of the PTWI from this exposure route. In India consumption of lead in chocolate products would account for approximately 28% of the PTWI for lead for the average child and exceed this threshold for many children.

A potentially important source of lead contamination in cocoa beans and cocoa is the tetra-ethyl lead (TEL) additive in gasoline, which is still common in many African countries (Nriagu 1992; Nriagu et al. 1996). For example, Nigerian gasoline contains 0.4–0.8 g/L lead, which is among the highest in the world (Ogunsola et al. 1994a); approximately 90% of the lead pollution in Nigeria is derived from the combustion of leaded gasoline, with total estimated annual lead aerosol emissions of 2,800 metric tons (Obioh et al. 1993). Those emissions are reflected in the contamination of Nigerian dusts, plants, and foods (Ajayi and Kamson 1983; Ndiokwere 1984; Nriagu 1992; Odukoya and Ajayi 1987; Ogunsola et al. 1994b; Onianwa and Egunyomi 1983) and the elevated blood lead concentrations noted in several studies of Nigerian people (Ademuyiwa et al. 2002; Nriagu 1992; Nriagu et al. 1997; Omokhodion 1994). This ongoing contamination from leaded gasoline emissions is consistent with the report by Bahemuka and Mubofu (1999) that lead concentrations of some foods grown on the African continent still exceed both WHO and the United Nation’s Food and Agricultural Organization permissible levels of 5 ng/g.

Unfortunately, the contribution of different natural and industrial leads in cocoa beans, cocoa, and chocolate products has not been resolved. To the best of our knowledge, there has been no systematic auditing of the sources of lead during the manufacture of chocolate products—from the harvesting of cocoa beans to the finished products. An objective of the present study was to determine the concentration and isotopic composition of lead in cocoa beans and soils from cocoa plantations of Nigeria to establish the baseline lead concentration of the beans and determine the relative contribution of soil lead to that concentration. Our other objective was to determine the contribution of other lead sources in cocoa bean products, cocoa, and chocolates, again using lead concentration and stable isotopic composition analyses.

## Materials and Methods

### Sample collection and preparation.

We collected cocoa bean and sediment samples in November and December 2002 from six farms in the three highest cocoa-producing states in Nigeria (Ondo, Osun, and Ogun), which were identified from statistics presented in the Central Bank of Nigeria Annual Reports (CBN 2002). Soil samples were collected at four depths in the soil profile: 0–10 cm, 0–20 cm, 35–50 cm, and 85–100 cm. At each farm, six separate samples were taken from the first and third profiles, and three samples were taken from the other profiles. Ripe cocoa bean samples were taken from each farm, as well as a sample of cocoa beans that had been fermented and dried in their shells, ready for export.

To create representative samples for each Nigerian farm, we made composites for each soil profile, both types of cocoa beans and the shells corresponding to the ripe cocoa beans collected. This process yielded homogenized composites of at least four soil, two cocoa bean, and two cocoa bean shell samples per farm for analysis. In addition to the Nigerian samples, cocoa beans from other countries and finished chocolate products including processed cocoa were collected for analysis.

All sample processing was conducted with established trace metal clean techniques (Scelfo and Flegal 2000) in trace metal clean rooms with HEPA (class 100) filtered air. Digestions in aqua regia (3:1 HCl:HNO_3_) were conducted using optima grade (Seastar Chemicals Inc., Sidney, British Columbia, Canada) reagents. Procedural blanks and reference materials from the National Institute of Standards and Technology [Standard Reference Material (SRM) 1547; NIST, Gaithersburg, MD, USA] and the National Research Council of Canada (MESS-3; Ottawa, Ontario, Canada) were digested concurrently with all samples to assess the efficacy of the method.

### Analysis of lead content.

We analyzed lead concentrations using the same protocols delineated for previous analyses of lead in calcium supplements (Scelfo and Flegal 2000), with a Finnegan MAT Element high-resolution magnetic sector inductively coupled plasma mass spectrometer (HR-ICPMS) (Thermo Electron Corporation, Waltham, MA). Lead concentrations were derived from instrumental scans of the three major lead isotopes (^206^Pb, ^207^Pb, and ^208^Pb) and that of bismuth (^209^Bi). The sum of intensities for the stable lead isotopes was normalized to the bismuth internal standard to correct instrumental variations in sensitivity. Percent recoveries of MESS-3 and SRM 1547 averaged 94% and 98%, respectively.

### Isotopic measurements.

In addition to the concentration measurements made with the HR-ICPMS, we made preliminary isotopic measurements from instrumental scans of all stable lead isotopes (^204^Pb, ^206^Pb, ^207^Pb, and ^208^Pb). Fractionation corrections were derived from concurrent analyses of SRM 981 (NIST; common lead standard reference material). Corrections for ^204^Pb/^206^Pb, ^207^Pb/^206^Pb, and ^208^Pb/^206^Pb averaged +0.006, +0.001, and +0.003, respectively.

We followed the initial HR-ICPMS measurements with thermal ionization mass spectrometry (TIMS) measurements of selected sample aliquots, using a VG Sector 54-WARP TIMS (GV Instruments, Wythenshawe, Manchester, England) and established protocols (Steding et al. 2000). Before these analyses, samples were dried and purified using Dowex AG1-X8 anion exchange resin (50–100 mesh) (Bio-Rad Labs, Hercules, CA) and high-purity (Seastar) hydrobromic acid. Soil samples were passed through the columns once, whereas other samples (cocoa bean shells, processed cocoa, and chocolate products) were passed through the columns a second time to further improve their purity and optimize their isotopic composition analyses. The eluates were dried and loaded onto rhenium filaments with a phosphoric acid and silica gel ionization enhancer. Procedural blanks were determined with a ^208^Pb spike and were < 0.1% of the samples analyzed. Fractionation corrections were calculated from concurrent analyses of SRM 981 using the linear law, with the mass bias per atomic mass unit correction averaging 0.0011 ± 0.0002. The precision of reproducibility for NIST 981 was ± 0.020 for ^206^Pb/^204^Pb, ± 0.0005 for ^206^Pb/^207^Pb, and ± 0.0009 for ^208^Pb/^207^Pb.

## Results and Discussion

As shown in Table 1, lead concentrations in cocoa beans ranged from ≤ 0.103 to 1.78 ng/g, with an average concentration of 0.512 ng/g. This average is comparable with the lowest reported concentrations of lead in food (Flegal et al. 1990; Tahvonen and Kumpulainen 1995). Moreover, that average is considered to be conservatively high because it uses the detection limit (0.103 ng/g) for three samples with lead concentrations below that limit and includes the relatively high (order-of-magnitude) concentration of another sample (Kango Village Farm; sample taken directly from husk) that appears to have been contaminated when compared with the concentrations of the other 11 samples. Therefore, we assumed that the average lead concentration of cocoa beans from the Nigerian farms is < 0.5 ng/g and may well be < 0.1 ng/g.

**Figure 1:**
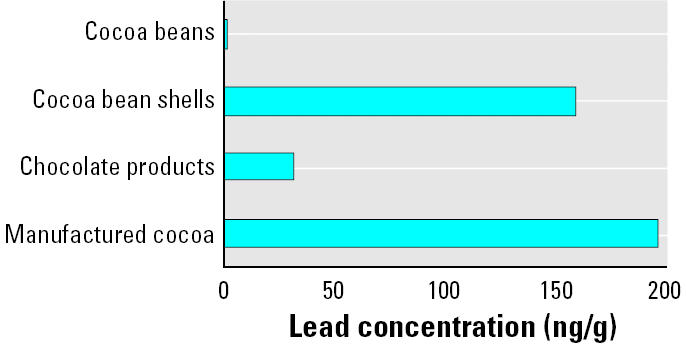
A comparison of average lead concentrations (ng/g) for analyzed cocoa beans, cocoa bean shells, chocolate products, and manufactured cocoa.

Although the lead content of cocoa beans is as low as or lower than those of hundreds of different foods in the United States and elsewhere, lead concentrations of manufactured cocoa are among the highest of all foods. The values, displayed in Table 2, are similar to the value (280 ng/g) independently determined by West Coast Analytical Service (WCAS; Santa Fe Springs, CA) for processed cocoa (Northington J, personal communication) and the range of values (140–297 ng/g) reported by Mounicou et al. (2002). More recently, Mounicou et al. (2003) reported lead concentrations in cocoa powder ranging from 11 to 769 ng/g, with an average of 255 ng/g. Our average concentration, 197 ng/g, about 3% of a child’s PTWI, is also comparable with the highest concentration reported for any food (boiled shrimp maximum, 210 ng/g) in the U.S. TDS (FDA 2000). Similarly, the average lead concentration for 23 chocolate products measured by WCAS, 32.5 ng/g, is indicative of contamination, and the individual values, displayed in Table 3, are similar to the mean (27 ng/g) reported in the U.S. TDS for a plain milk chocolate bar (U.S. FDA 2000). Most notably, the average lead concentration of those chocolate products is approximately 60-fold higher than the average lead concentration of the Nigerian cocoa beans. A comparison of lead concentrations in the analyzed source material and the finished products is shown in Figure 1.

Although the lead content of cocoa beans is as low as or lower than those of hundreds of different foods in the United States and elsewhere, lead concentrations of manufactured cocoa are among the highest of all foods. The values, displayed in Table 2, are similar to the value (280 ng/g) independently determined by West Coast Analytical Service (WCAS; Santa Fe Springs, CA) for processed cocoa (Northington J, personal communication) and the range of values (140–297 ng/g) reported by Mounicou et al. (2002). More recently, Mounicou et al. (2003) reported lead concentrations in cocoa powder ranging from 11 to 769 ng/g, with an average of 255 ng/g. Our average concentration, 197 ng/g, about 3% of a child’s PTWI, is also comparable with the highest concentration reported for any food (boiled shrimp maximum, 210 ng/g) in the U.S. TDS (FDA 2000). Similarly, the average lead concentration for 23 chocolate products measured by WCAS, 32.5 ng/g, is indicative of contamination, and the individual values, displayed in Table 3, are similar to the mean (27 ng/g) reported in the U.S. TDS for a plain milk chocolate bar (U.S. FDA 2000). Most notably, the average lead concentration of those chocolate products is approximately 60-fold higher than the average lead concentration of the Nigerian cocoa beans. A comparison of lead concentrations in the analyzed source material and the finished products is shown in Figure 1.

**Figure 2:**
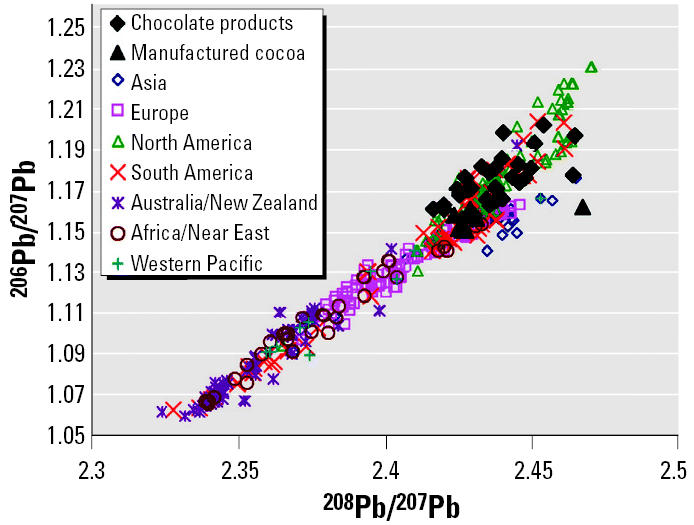
Isotopic compositions of analyzed chocolate products and manufactured cocoa compared with those in global aerosols measured by Bollhöfer and Rosman (2000, 2001, 2002).

Possible origins of contaminant lead in both the manufactured cocoa and chocolate products are identified by their lead isotopic ratios, which are also shown in Tables 2 and 3. Figure 2 provides a comparison of the lead isotopic ratios for chocolate products and manufactured cocoa with the ratios of the world’s industrial aerosols, as compiled by Bollhöfer and Rosman (2000, 2001, 2002). The plot shows that isotopic compositions of all of the chocolate products overlap with those of lead aerosols measured by Bollhöfer and Rosman (2000, 2001, 2002), but the isotopic compositions of manufactured cocoa and chocolate products are variable. Consequently, there is no single, identifiable source of contaminant lead in either processed cocoa or chocolate products, which is consistent with reports of geographic differences of lead concentrations in cocoa powder (Mounicou et al. 2003).

One of those sources may be cocoa bean shells, which have been shown to be very efficient in removing lead from solutions (Meunier et al. 2003a, 2003b, 2003c, 2004). Meunier et al. (2003a, 2003b) showed extraneous lead adsorption onto cocoa bean shells of up to 17,000 μg/g, or approximately 35 million times greater than our conservatively high calculation of the average lead concentration in cocoa beans. The shells can thus be regarded as an excellent protective shield against intrusion of lead into the bean from external sources before the beans are harvested. Furthermore, the removal of lead results in an increase in solution pH (removal of protons) and the release of calcium, magnesium, potassium, and sodium from the cocoa shells (Meunier et al. 2003c). The modification of ion balance may result in the transfer of lead from the bean to the shell, a decontamination process. Because of their capacity to scavenge lead, the shells may become a source of contamination after the beans are harvested.

That potential is indicated by the high lead concentrations of cocoa bean shells listed in Table 4. The average of our measurements of lead in cocoa bean shells (160 ng/g) is approximately 320-fold greater than the average values of lead measured in Nigerian cocoa beans (Figure 1). The disparity between lead levels in the cocoa beans and shells is consistent with the literature on lead contamination in foods, which have shown that contamination is greatest on plant surfaces that are subject to the direct deposition of industrial lead aerosols. For example, two decades ago it was determined that lead concentrations of spinach in the United States were elevated 30-fold from a baseline value of 0.0015 μg/g to 0.045 μg/g by atmospheric depositions of industrial lead, whereas the lead concentrations of peanuts were only elevated 2-fold from a baseline value of 0.005 μg/g to 0.010 μg/g from atmospheric contamination (Flegal et al. 1990). This potential source of contamination is further evidenced by the absence of a measurable increase in lead concentration between the beans sampled directly after removal from the husk of the plant and those that had been fermented in banana and plantain leaves and sun-dried (Oke Osun Farm; directly from husk, 0.846 ng/g; fermented and dried, 0.839 ng/g).

**Figure 3:**
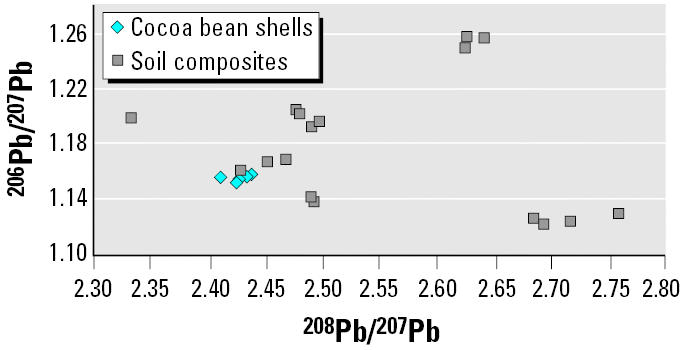
Isotopic compositions of cocoa bean shells compared with those of soil profile composites.

The presence of contaminant lead in bean shells from the cocoa farms is substantiated by the concentrations we observed in the various soil profile composites of this study (Table 5). The average lead concentration was 14.2 μg/g, which is consistent with the survey of Chukwuma (1997), who reported the lowest lead value measured in Nigerian soils as 10 μg/g. The lead isotopic ratios of the soil profiles, listed in Table 6 and displayed in Figure 3, are variable, indicating that multiple sources (e.g., historically different sources of TEL, pesticides, fertilizers, machinery) are responsible for the contamination observed. Although the isotopic area encompassed by the soil profiles overlaps the lead isotopic compositions of the cocoa bean shells and may be a source of current contamination, as shown in Figure 3, the similarities in the isotopic compositions of cocoa bean shells are indicative of a single predominant source of lead contamination at the cocoa farms. In light of the numerous published reports on the predominance of gasoline emissions as a source of lead contamination in Nigeria, this source is tentatively attributed to TEL, although we were not able to obtain samples of Nigerian gasoline for isotopic composition analysis in the United States for this study.

**Figure 4:**
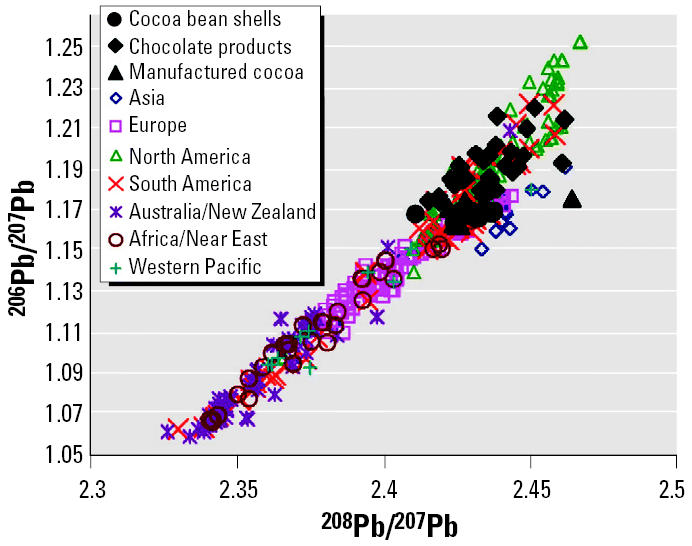
Isotopic compositions of cocoa bean shells compared with those of chocolate products and manufactured cocoa.

As shown in Figure 4, lead isotopic ratios of cocoa bean shells overlap those of manufactured cocoa and chocolate products. Because the FDA limits cocoa bean shells to comprise a maximum of 1.75% in finished chocolate products (Beckett 2002), lead concentrations observed for cocoa bean shells are too low to account for the contamination observed in the finished products. Coupling the previously discussed capacity for cocoa bean shells to adsorb lead with their lead isotopic composition suggests that further contamination of cocoa bean shells during the fermentation and drying stages at the farm is a possible source of some of the contaminant lead observed in the finished products. However, the larger spread of lead isotopic compositions for the manufactured cocoa and chocolate products indicates other sources of contamination occurring after the cocoa farms. Further studies investigating bean storage and intermediate phases of shipping and processing are needed to isolate the predominant source of lead found in the chocolate and cocoa products.

In summary, chocolate products and manufactured cocoa contain relatively high levels of contaminant lead compared with the baseline value for Nigerian cocoa beans used to make those products. Isotopic composition analyses of the products indicate multiple sources of contamination of industrial origin, which is consistent with the observation that there are numerous sources of lead contamination during the production of cocoa that have yet to be identified (COPAL 2004a, 2004b). Similar lead isotopic composition in contaminated cocoa bean shells from Nigeria, together with the high ability of cocoa bean shells to adsorb lead, suggest that contamination during cocoa processing at each farm may be responsible for some of the contamination in cocoa products; we propose that the ongoing use of leaded gasoline in Nigeria contributes to that contamination. However, the low lead concentration in cocoa beans compared with those of manufactured cocoa and chocolate products indicates that most lead contamination in those products occurs after the beans are harvested and dried, during the shipping of those beans and/or the manufacturing of cocoa and chocolate products.

## Figures and Tables

**Table 1:** Lead concentrations (ng/g) of cocoa beans taken directly from husk and after being fermented and dried at Nigerian farms.

		Directly from husk		Fermented and dried	
State	Farm	[Pb]	% RSD	[Pb]	% RSD
Ogun	Oke Osun, Ibese	0.846	9.81	0.839	7.73
	Kango Village	1.78	62.4	0.941	3.83
Ondo	Igbo Eleruku, Ita Ogbolu	0.213	5.16	0.182	4.45
	Ase Igbo	—	—	< DL	7.06
Osun	Idi Obi I	0.313	8.30	< DL	10.1
	Aba Arawense, Modakeke	< DL	8.06	0.211	9.51

Abbreviations: % RSD, percent relative standard deviation, reported as the internal error (σ) in the HR-ICPMS measurements; DL, detection limit (0.103 ng/g).

**Table 2:** Lead isotopic ratios and concentrations (ng/g) of manufactured cocoa.

Sample	^206^Pb/^207^Pb	^208^Pb/^207^Pb	[Pb]	% RSD
Baking chocolate 1A	1.149 (4)	2.428 (4)	251	11.8
Baking chocolate 1B	1.148 (2)	2.425 (9)	241	0.75^a^
Baking chocolate 1C	1.150 (5)^b^	2.426 (8)^b^	263	0.75^a^
Baking chocolate 2A	1.160 (6)	2.429 (5)	188	6.93
Baking chocolate 2B	1.160 (1)	2.423 (1)	200	0.4^a^
Baking chocolate 2C	—	—	181	0.5^a^
Cocoa powder 1A	1.158 (3)	2.431 (11)	188	5.17
Cocoa powder 1B	—	—	186	0.8^a^
Cocoa powder 1C	—	—	183	0.7^a^
Cocoa powder 2	1.183 (3)	2.467 (1)	147	28.2

% RSD, percent relative standard deviation. Except where noted, isotopic compositions from multiple digests and analyses were averaged, and numbers in parentheses are the error (2σ) of these averages. % RSD is the error (2σ) from multiple analyses except where noted.

a Reported as the internal error (2σ) from the HR-ICPMS counting statistics.

b Reported as the internal error (2σ) calculated from the average RSD from concurrent SRM 981 analyses on the HR-ICPMS.

**Table 3:** Lead isotopic ratios and concentrations (ng/g) of chocolate products.

Sample	[Pb]	^206^Pb/^207^Pb	^208^Pb/^207^Pb
Bittersweet chocolate 1	69.8	1.1712 (2)	2.4282 (1)
Bittersweet chocolate 2	29.4	1.1797 (1)	2.4357 (1)
Chocolate candy 1	23.0	1.2029 (1)	2.4542 (1)
Chocolate candy 2	27.9	—	—
Chocolate candy 3	18.7	1.1716 (1)	2.4242 (2)
Chocolate candy 4	20.0	—	—
Chocolate candy 5	11.9	—	—
Chocolate pudding 1	14.8	—	—
Chocolate pudding 2	15.9	—	—
Dark chocolate 1	49.6	1.1768 (1)	2.4276 (1)
Dark chocolate 2	40.9	1.1774 (1)	2.4271 (1)
Dark chocolate 3	57.6	1.1688 (1)	2.4252 (1)
Dark chocolate 4	29.4	1.1992 (3)	2.4405 (2)
Dark chocolate 5	26.0	—	—
Dark chocolate 6	35.1	1.1866 (1)	2.4400 (1)
Milk chocolate 1	23.4	1.1617 (1)	2.4201 (1)
Milk chocolate 2	14.9	1.1619 (8)	2.4163 (5)
Semisweet chocolate 1	42.1	1.1720 (1)	2.4294 (1)
Semisweet chocolate 2	41.7	1.1826 (1)	2.4330 (1)
Semisweet chocolate 3	31.1	1.1638 (1)	2.4198 (1)
Semisweet chocolate 4	56.1	1.1836 (11)	2.4456 (12)
Semisweet chocolate 5	36.2	1.1766 (1)	2.4275 (1)
Semisweet chocolate 6	—	1.1820 (5)	2.4376 (4)

Numbers in parentheses are the error (2σ) recorded from TIMS counting statistics. Isotopic ratios were obtained at the University of California, Santa Cruz, and concentrations were determined at WCAS.

**Table 4:** Lead concentrations (ng/g) and isotopic compositions of cocoa bean shells from Nigerian farms.

State	Farm	Sample	^206^Pb/^207^Pb	^208^Pb/^207^Pb	[Pb]	% RSD
Ogun	Oke Osun, Ibese	Shell 1	1.155 (5)	2.434 (9)	61	1.92
		Shell 2	1.156 (5)	2.412 (8)	72	0.28
	Kango Village	Shell 1	1.158 (5)	2.439 (9)	74	0.75
		Shell 2	1.156 (5)	2.438 (9)	82	0.24
Ondo	Igbo Eleruku, Ita Ogbolu	Shell 1	1.1566 (1)^a^	2.4336 (2)^a^	417	0.46
		Shell 2	1.153 (5)	2.425 (9)	409	0.22
	Ase Igbo	Shell 1	1.158 (5)	2.436 (9)	73	0.46
		Shell 2	1.158 (5)	2.431 (9)	144	0.29
Osun	Idi Obi I	Shell 1	1.156 (5)	2.428 (9)	185	0.22
		Shell 2	1.155 (5)	2.432 (9)	132	0.26
	Aba Arawense, Modakeke	Shell 1	1.155 (5)	2.434 (9)	120	0.24
		Shell 2	1.156 (5)	2.429 (9)	157	0.18

% RSD, percent relative standard deviation. Except where noted, numbers in parentheses are the internal error (2σ) calculated from the average relative deviation from concurrent analyses of SRM 981 on the HR-ICPMS. The percent relative deviation is reported as the internal error (σ) from the HR-ICPMS counting statistics.

a Isotopic compositions and the error (2σ) are from TIMS analysis.

**Table 5:** Lead concentrations (μg/g) of soil profile composites from Nigerian farms.

		0–10 cm		0–20 cm		35–50 cm		80–100 cm	
State	Farm	[Pb]	% RSD	[Pb]	% RSD	[Pb]	% RSD	[Pb]	% RSD
Ogun	Oke Osun, Ibese	3.54	2.4	3.00	0.7	2.94	0.6	3.46	0.7
	Kango Village	17.0	0.5	21.5	0.4	40.6	0.5	29.8	0.4
Ondo	Igbo Eleruku, Ita Ogbolu	11.2	2.2	12.1	1.2	12.6	0.4	—	—
	Ase Igbo	30.8	0.6	17.4	0.7	25.5	0.7	17.7	0.4
Osun	Idi Obi I	10.8	2.3	11.7	0.4	10.6	0.4	14.9	0.4
	Aba Arawense, Modakeke	7.55	1.3	6.86	2.4	6.25	0.5	8.21	0.4

% RSD, percent relative standard deviation reported as the internal error (σ) from HR-ICPMS counting statistics.

**Table 6:** Lead isotopic compositions of soil profile composites from Nigerian farms.

		0–10 cm		0–20 cm		35–50 cm		80–100 cm	
State	Farm	^206^Pb/^207^Pb	^208^Pb/^207^Pb	^206^Pb/^207^Pb	^208^Pb/^207^Pb	^206^Pb/^207^Pb	^208^Pb/^207^Pb	^206^Pb/^207^Pb	^208^Pb/^207^Pb
Ogun	Oke Osun	1.168 (5)^a^	2.469 (9)^a^	1.1226 (1)^b^	2.6942 (2)^b^	1.1953 (1)^b^	2.4988 (1)^b^	1.1959 (1)^b^	2.4972 (1)^b^
	Kango Village	1.130 (5)^a^	2.760 (10)^a^	—	—	1.1237 (1)^b^	2.7179 (2)^b^	1.1259 (1)^b^	2.6860 (1)^b^
Ondo	Igbo Eleruku	1.138 (5)^a^	2.493 (9)^a^	1.141 (5)^a^	2.492 (9)^a^	—	—	—	—
	Ase Igbo	1.2558 (1)^b^	2.6417 (2)^b^	1.161 (5)^a^	2.428 (9)^a^	1.2561 (1)^b^	2.6269 (2)^b^	1.2493 (1)^b^	2.6248 (4)^b^
Osun	Idi Obi I	1.1922 (1)^b^	2.4911 (6)^b^	—	—	1.1983 (1)^b^	2.3329 (1)^b^	1.1671 (1)^b^	2.4527 (1)^b^
	Aba Arawense	1.203 (5)^a^	2.479 (9)^a^	1.200 (5)^a^	2.482 (9)^a^	—	—	—	—

a Numbers in parentheses are the internal error (2σ) calculated from the average relative standard deviation from concurrent SRM 981 analyses on the HR-ICPMS.

b Numbers in parentheses are the internal error (2σ) from the TIMS counting statistics.
